# Orbital volume, ophthalmic sequelae and severity in unilateral coronal synostosis

**DOI:** 10.1007/s00381-021-05065-3

**Published:** 2021-02-10

**Authors:** Sophia A. J. Kronig, Otto D. M. Kronig, Marcel Zurek, Léon N. A. Van Adrichem

**Affiliations:** 1 Department of Plastic and Reconstructive Surgery and Hand Surgery, University Medical Centre Utrecht, Heidelberglaan 100, 3584 CX Utrecht, The Netherlands; 2 Department of Ophthalmology, Erasmus University Medical Centre (Erasmus MC), Rotterdam, The Netherlands

**Keywords:** Quantification, Anterior plagiocephaly, Orbital volume, Synostosis, Ophthalmic sequelae

## Abstract

**Purpose:**

Unilateral coronal synostosis (UCS) results in an asymmetrical skull, including shallow and asymmetrical orbits, associated with reduced orbital volume and high prevalences of ophthalmic sequelae. Aim is to link orbital volumes in patients with UCS to severity according to UCSQ (Utrecht Cranial Shape Quantifier) and presence of ophthalmic sequelae.

**Methods:**

We included preoperative patients with UCS (≤ 18 months). Orbital volume was measured on CT scans by manual segmentation (Mimics software (Materialise, Leuven, Belgium)), and severity of UCS was determined by UCSQ. Orbital volume of affected side was compared to unaffected side using Wilcoxon signed rank test. Orbital volume ratio was calculated (affected/unaffected volume) and compared to the category of UCSQ by Kruskal-Wallis test. Opthalmic sequelae were noted.

**Results:**

We included 19 patients (mean age 7 months). Orbital volume on affected side was significantly lower (*p* = 0.001), mean orbital volume ratio was 0.93 (SD 0.03). No significant differences in group means of orbital volume ratio between different levels of severity of UCSQ were found (Kruskal-Wallis *H* (2) = 0.873; *p* > 0.05). Ophthalmic sequelae were found in 3 patients; one had adduction impairment and strabismus (mild UCS), one had astigmatism (moderate UCS), and one had abduction impairment (on both ipsi- and contralateral side) and vertical strabismus (severe UCS).

**Conclusion:**

No association between orbital volume ratio and severity of UCS was found. Side-to-side asymmetry in orbital volume was noted. No association between either preoperative orbital volume ratio or severity of UCS and the presence of preoperative ophthalmic sequelae was found.

## Introduction

Unilateral coronal synostosis (UCS) or anterior plagiocephaly is a result of synostosis of a unilateral coronal suture. In general, the prematurely closed coronal suture results in restriction of growth of the normal skull, brain and face, leading to a deformed skull and midface hypoplasia, including shallow orbits and an asymmetry between the orbits. The shape of the orbit on the side of the fused suture is compromised; the supraorbital rim is shifted backward upward; this is called the harlequin orbit [4, 17, 24]. The visible orbital dysmorphology in patients with UCS is associated with a reduction in orbital volume [3, 5, 7].

Additionally, in patients with UCS, high prevalences of ophthalmic problems are found, which are thought to be secondary to the anatomical deformities in the orbit on the ipsilateral side, and the resulting orbital asymmetry may additionally underlie visual abnormalities [2, 21, 31]. These ophthalmic sequelae include impairment of eye movement, strabismus, amblyopia, astigmatism and visual field defects, and may occur on both the ipsi- and contralateral side of the synostosis [2, 5–7, 12, 14, 21–23, 31].

Nevertheless, currently little literature is available regarding the correlation between the severity of the deformity of UCS and the (altered) orbital volumes [7]. Also, no literature is present regarding the presence of ophthalmic sequelae and the severity of UCS and the orbital volume.

Recently, a novel method for quantification of severity of UCS was introduced, UCSQ (Utrecht Cranial Shape Quantifier) [18]. This outline-based method of quantification of skull shape deformities has the advantage of capturing the geometric skull shape variation. External landmarks (soft tissue landmarks, visible with the bare eye) are used to determine a reference plane at 4-cm height on CT (computed tomography) scan. Following, an algorithm measures distance and angle from centre of mass on the plane to the skull outline, leading to sinusoid curves. These curves demonstrate an occipital peak, a left and right lateral trough and a central frontal peak. The resulting curves are specific and characteristic for unilateral coronal synostosis [19, 20]. Furthermore, UCSQ is proven to be suited for quantification of severity of UCS by using two characteristic variables: asymmetry ratio of frontal peak and ratio of frontal peak gradient [18].

The aim of the present study is to link calculated orbital volumes in preoperative patients with UCS to the degree of severity of UCS according to UCSQ and to the presence of ophthalmic sequelae.

## Material and methods

### Patients

For the purposes of this study, we included preoperative children (age ≤ 18 months) with CT confirmed UCS. These patients were diagnosed at the Erasmus Medical Centre, Sophia Children’s Hospital Rotterdam.

To be eligible for inclusion, the preoperative CT scan needed to contain both orbits and the whole skull. Any subject with additional synostosis, other craniofacial abnormality or (orbital or cranial) surgery prior to the first available CT scan were excluded. The CT scans used for the purposes of this study were part of the routine diagnostic evaluation in patients with a suspected craniosynostosis. The slice thickness of the CT scans needed to be less than 3.00 mm.

The study was approved by the local Medical Ethics Review Committee (MEC-2016-467). The study was deemed a retrospective clinical study and did not require formal research ethics approval under the Medical Research Involving Human Subjects Act.

Patient characteristics were noted, including the need for sedation during CT scanning. Complete or incomplete closure of the coronal suture and involvement of the squamous and sphenofrontal sutures were noted.

### Calculating the orbital volume

We used the term ‘affected side’ to characterise the side of premature fusion of the coronal suture and ‘unaffected side’ refers to the absence of premature closure of the coronal suture. Left- and right-sided anterior plagiocephaly is considered one patient group, using the subdivision of affected and unaffected side.

**Fig. 1:**
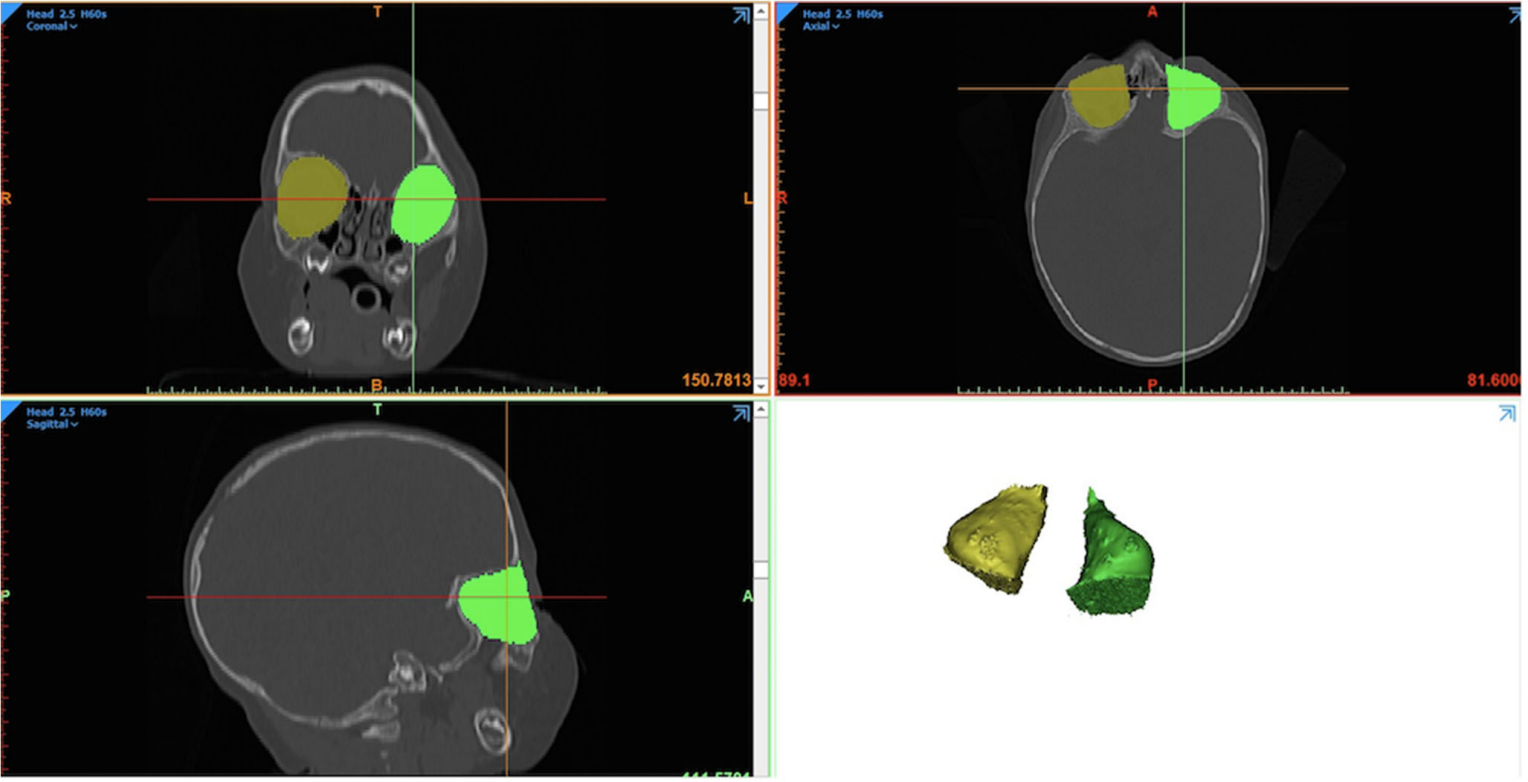
Orbital boundaries in Mimics and the resulting three-dimensional orbital model

The software program Mimics (21.0, Materialise, Leuven, Belgium) was used to import and analyse the DICOM (Digital Imaging and Communications in Medicine) data from CT scans. In order to outline the interface between the bony walls and soft tissues in the orbital cavity, a mask was created using a threshold of − 240 to 226 HU [13]*.* This mask enclosed the intraorbital soft tissues, but excluded the bony boundaries. The anterior and posterior boundaries are as described by Nout et al. [28]. The anterior boundary was defined as a straight line connecting the most antero-inferior point of the supraorbital rim and the most antero-superior point of the infraorbital rim in the sagittal plane. The posterior boundary was defined as the anterior segment of the optical canal, thus excluding the optic canal from volume calculations. The remaining orbital boundaries (superior, inferior, medial and lateral) were defined by the bony structures of the orbit; in case of bony interruptions or thin bony walls, a straight line was drawn between the nearest bony boundaries. On each sagittal slice of the CT scan, these boundaries of each orbit were manually outlined. Measurements were performed by one (experienced) examiner. Following, the orbital volume was automatically calculated from the 3D models of the manually segmented orbit (Fig. 1).

Orbital volume of five randomly selected patients was remeasured by both the first examiner and a second (experienced) examiner, in order to assess consistency and inter-rater reliability.

### Classification of severity

**Fig. 2:**
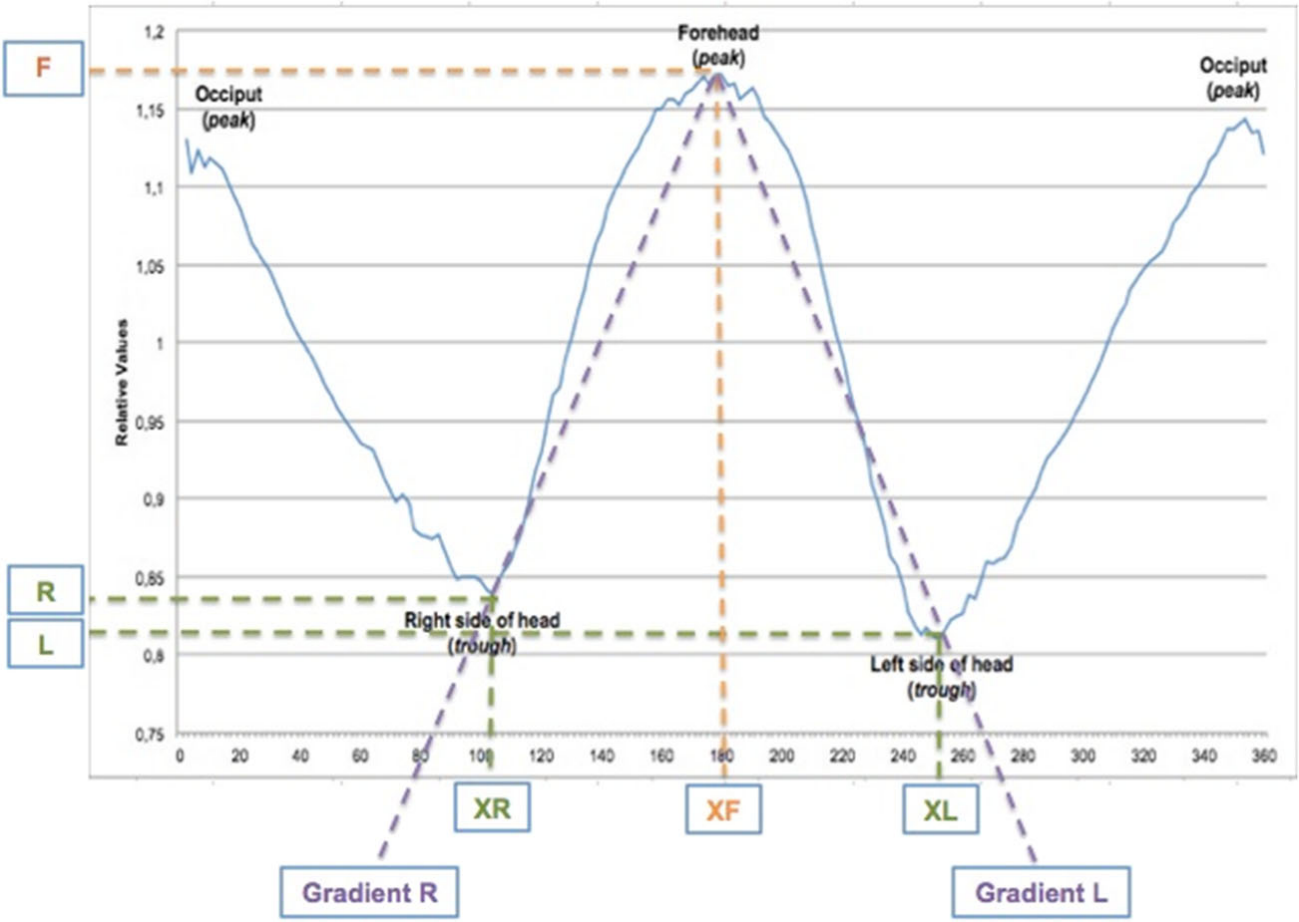
Visualization of the used variables. *F*, Maximum of forehead; *L*, minimum value of left side of the head; *R*, minimum value of right side of the head; *XF*, *X* value of maximum forehead value; *XL*, *X* value of the minimum value of the width on the left side; *XR*, *X* value of the minimum value of the width on the right side; *Gradient L* and *R*, Δ*Y/*Δ*X*, where Δ*Y = F − R* and/or *F − L*, and Δ*X = XF − XR* and/or *XL − XF*

Severity of UCS can be assessed by UCSQ for UCS. UCSQ uses the following variables: asymmetry ratio of frontal peak (left-sided UCS: (XL-XF)/(XF-XR); right-sided UCS: (XF-XR)/(XL-XF)) and ratio of frontal peak gradient (gradient affected side/gradient unaffected side) (Fig. 2). A good correlation was found between severity of UCS and these combined variables [18].

Figure 2 shows an example of an obtained curve. The curve starts at the occiput, and skull outline is followed clockwise. After the first peak, resembling the occiput, the curve decreases, because the distance from the centre of mass to the right side of the head is shorter than the distance from centre of mass to the forehead or occiput. The second peak resembles the forehead; again, the curve decreases to the left side of the head and increases to the occiput.

In order to differentiate between the different levels of severity of UCS, we used the most distinctive variables for UCS, namely the aforementioned: asymmetry ratio of frontal peak and ratio of gradient. A plagiocephalic skull is skewed compared to the normal skull. Therefore, the difference between the mean values of a control skull for the previous variables and those of a patient with UCS is indicative for severity. We used the mean values from the control patients, as reported in our previous study [19].

The following calculation to determine cutoff values and the different classes of severity (mild, moderate, severe) was developed: (asymmetry ratio of frontal peak – 1.067) × − 0.23 + (ratio of gradient – 0.90) × 0.57). In this calculation the values 1.067 and 0.90 are the mean values of the variables (asymmetry ratio of frontal peak and ratio of gradient respectively) in control patients. In the calculation, the differences between the variables in patients with UCS and control patients are multiplied (by − 0.23 and 0.57) in order to give each variable the same weight in the resulting outcome. Following, cutoff values for each subgroup of severity were proposed: mild ≥ − 0.1, moderate − 0.1 – − 0.5, severe ≤ − 0.5.

### Additional parameters

The following parameters were measured, calculated or reported based on (3D)-CT scan: orbital index (OI), proptosis, presence of deviation of nasal root and angulation of the sphenoid ridge.

Height and width of ipsilateral and contralateral orbits relative to the synostosis were measured. Height was measured from a vertical line dropped from the lateral extent of the supraorbital notch to the infraorbital rim; width was measured as a horizontal line extending from the zygomaticofrontal suture to the medial orbital rim. OI was defined as the ratio between the orbital width and orbital height [10, 33].

Proptosis is calculated on the axial plane; a reference line for measurement is drawn, the interzygomatic line (a line between the anterior portions of the zygomatic bones). Following, the distance from this line to the anterior surface of the globe is measured, and should be < 23 mm [15].

Presence of nasal root deviation was assessed from both the axial and frontal planes.

Angulation of the sphenoid ridge was measured on the axial plane. A tangential line passing through the lesser wing of the sphenoid was drawn. Following, the point of intersection between both a line from the tip of the anterior clinoid process and a line from the terminal point of the lateral extension of the sphenoid wing on the lateral wall of the middle cranial fossa was established. The angle between these two lines was measured and noted as the angulation of the sphenoid ridge [16].

### Ophthalmic sequelae

Preoperative medical records were searched for the following ophthalmic data: impairment of eye movement, strabismus, amblyopia, astigmatism and visual field defects. The smooth pursuit of eye movement that was used to determine the presence of impairment of eye motility, direction of impairment, and affected eye was noted. Presence and direction of strabismus was noted. Presence and affected eye of amblyopia, astigmatism and visual field defects were noted. Prevalence of ophthalmic sequelae was calculated.

### Statistical analysis

Data regarding intra- and interrater reliability were analysed with intraclass correlation coefficients (ICC) with acceptable reliability criteria > 0.75 [29].

For comparison of the orbital volumes of the affected and unaffected sides within patients, we used Wilcoxon signed rank test. Additionally, the ratio between the orbital volume on the affected and unaffected side (orbital volume ratio) was calculated.

One-way analysis of variance (ANOVA) or Kruskal-Wallis test was used to compare orbital volume ratio to category of UCSQ. The used test was based on normality of data.

Based on the sample size of the patients with ophthalmic sequelae, descriptive statistics of the ophthalmic sequelae were noted.

Statistical analyses were performed using the Statistical Package for the Social Sciences (SPSS) for Windows (Version 21, SPSS Inc., Chicago, IL, USA). Statistical significance was set at a *p* value ≤ 0.05.

## Results

We included 19 children with UCS. Demographics can be found in Table 1. Slice thickness of CT was 1.25 mm in all patients with UCS (high-resolution CT); 13 of the 19 patients (64.4%) were sedated during CT scanning. The coronal suture was completely prematurely closed in 18 of the 19 patients (94.7%). In 3 of the 19 patients, the squamous suture was closed (15.8%) and in 8 of the 19 patients (42.1%), the sphenofrontal suture was closed.

**Table 1:** Demographics and orbital volume

	UCS
Number of subjects	19
Sex	
Female	11 (57.9%)
Male	8 (42.1%)
Age at CT *(*months) (mean (min.–max.))	7 (1–18)
Side	
Right	8 (42.1%)
Left	11 (57.9%)
Orbital volume of affected side (cm^3^) (mean (SD))	13.10 (1.74)
Orbital volume of unaffected side (cm^3^) (mean (SD))	14.17 (1.97)
Ratio of affected to unaffected side (mean (SD))	0.93 (0.03)

*SD* standard deviation

### Orbital volume

Intra-rater (0.80) and inter-rater (0.95) reliabilities were found to be acceptable.

Mean calculated orbital volumes and orbital volume ratios can be found in Table 1. Table 2 shows the orbital volumes and orbital volume ratios per patient. In 100% of the UCS cases, the orbital volume on the affected side was smaller than on the unaffected side.

**Table 2:** Orbital volumes, severity and ophthalmic sequelae per patient

	Gender	Age (m)	OV affected side (cm^3^)	OV unaffected side (cm^3^)	OV ratio	UCSQ class of severity	Ophthalmic sequelae
1.	F	11	15.58	16.96	0.92	Mild	-
2.	M	4	11.15	11.99	0.93	Mild	-
3.	M	3	13.53	14.05	0.96	Mild	-
4.	M	1	9.97	11.13	0.90	Mild	-
5.	F	7	13.46	13.67	0.98	Severe	-
6.	F	7	13.61	14.66	0.93	Severe	-
7.	M	4	12.72	14.94	0.85	Mild	-
8.	F	8	14.79	16.33	0.91	Severe	-
9.	M	6	13.31	14.63	0.91	Moderate	-
10.	F	6	13.51	14.27	0.95	Moderate	-
11.	F	4	10.45	10.70	0.98	Mild	-
12.	F	6	14.02	15.04	0.93	Severe	-
13.	M	6	11.34	11.93	0.95	Severe	-
14.	F	9	13.64	14.82	0.92	Mild	Adduction impairmen*t* affected eye; Strabismus
15.	M	6	13.69	14.86	0.92	Moderate	-
16.	M	5	11.88	13.08	0.91	Moderate	Astigmatism
17.	F	18	15.88	17.51	0.91	Moderate	-
18.	F	10	15.40	16.63	0.93	Severe	-
19.	F	3	10.91	12.01	0.91	Severe	Abduction impairment both eyes; Strabismus (vertical)

*M*, male; *F*, female; *OV*, orbital volume; *UCSQ*, Utrecht Cranial Shape Quantifier

Side-to-side asymmetry in orbital volume was found in patients with UCS; the orbital volume on the affected side was found to be significantly lower than orbital volume on the unaffected side (*p* = 0.001).

### Orbital volume and severity of UCS

Mean of the calculation for severity of UCS ((asymmetry ratio of frontal peak – 1.067) × − 0.23 + (ratio of gradient – 0.90) × 0.57) was − 0.25 (− 0.69 − 0.77). Mean ‘Asymmetry ratio of frontal peak – 1.067’ was 0.59 (− 0.57 − 1.63) and mean ‘Ratio of gradient – 0.90’ was − 0.21 (− 0.61 − 1.12).

Based on the aforementioned classification of severity (mild ≥ − 0.1, moderate − 0.1 − − 0.5, severe ≤ − 0.5), 4 patients were categorized as mild, 12 as moderate and 3 as severe. Table 2 shows the class of severity of UCS per patient.

Kruskal-Wallis test was conducted to examine the differences in group means of orbital volume ratio between the different categories of UCSQ classification; no significant differences were found (Kruskal-Wallis *H* (2) = 0.873; *p* > 0.05).

### Additional parameters

Mean OI synostotic side was 1.1 (0.8–1.2) and mean OI nonsynostotic side was 1.0 (0.8–1.2). Mean proptosis on the synostotic side was 12 mm (9–17 mm) and on the nonsynostotic side, the mean was 12 mm (7–17 mm), all proptosis measurements were < 23 mm. Deviation of the nasal root was present in 15 of the 19 patients (78.9%). Mean angulation of the sphenoid ridge on the synostotic side was 116° (93–144°), and mean angulation on the nonsynostotic side was 118° (91–150°).

### Ophthalmic sequelae

Impairment of eye movement was noted in 2 patients (2/19; 10.5%); one impairment of adduction on the ipsilateral (affected) side and one impairment of abduction on both the ipsi- and contralateral side. The same two patients (2/19; 10.5%) had strabismus; one vertical strabismus (ipsilateral side) and one not noted. Astigmatism was found in 1 patient (1/19; 5.3%). No amblyopia and visual field defects were present.

The sample size of patients with ophthalmic sequelae was too small to apply statistical tests. All patients with ophthalmic sequelae had an orbital volume ratio within the second quartile of orbital volume ratio (less severe orbital volume difference).

The patient with adduction impairment and strabismus had mild UCS according to UCSQ; the patient with astigmatism had moderate UCS, and the patient with abduction impairment (on both the ipsi- and contralateral side) and vertical strabismus had severe UCS (Table 2).

The patient with adduction impairment and strabismus had complete closure of the coronal suture with an open squamous suture and a closed sphenofrontal suture. The OI on the synostotic side was 1.0 and 1.1 on the nonsynostotic side. Proptosis on both sides was 12 mm. The nasal root was deviated, and the angulation of the sphenoid ridge was 113° on the synostotic side and 119° on the nonsynostotic side.

The patient with astigmatism had complete closure of the coronal suture with an open squamous suture and a closed sphenofrontal suture. The OI on the synostotic side was 1.1 and 0.9 on the nonsynostotic side. Proptosis was 9 mm on the synostotic side and 11 mm on the nonsynostotic side. The nasal root was deviated, and the angulation of the sphenoid ridge was 144° on the synostotic side and 150° on the nonsynostotic side.

The patient with abduction impairment and vertical strabismus had complete closure of the coronal suture with open squamous and sphenofrontal sutures. The OI on the synostotic side was 1.1 and 0.9 on the nonsynostotic side. Proptosis was 13 mm on the synostotic side and 12 mm on the nonsynostotic side. The nasal root was deviated, and the angulation of the sphenoid ridge was 108° on the synostotic side and 127° on the nonsynostotic side.

## Discussion

Aim of the present study was to link calculated orbital volumes in preoperative patients with UCS to the degree of severity according to UCSQ and the presence of ophthalmic sequelae.

Based on our calculation of severity of anterior plagiocephaly ((asymmetry ratio of frontal peak – 1.067) × − 0.23 + (ratio of gradient – 0.90) × 0.57)), we proposed the following cutoff values in order to classify severity: mild ≥ − 0.1, moderate − 0.1 − − 0.5, severe ≤ − 0.5. However, these cutoff values are only based on 19 patients and further validation is needed in future research. For quantification by using UCSQ, we selected the following two variables: asymmetry ratio of frontal peak and ratio of gradient. Asymmetry ratio of frontal peak represents the shifting of the forehead, and ratio of gradient represents the asymmetry in flattening/abruptness of the forehead. By analysing curves of different craniosynostosis patient groups, we found these two variables most distinctive for both the diagnosis of UCS, as well as the severity of it.

Our study showed side-to-side asymmetry in orbital volume in preoperative patients with UCS, with a significantly lower orbital volume on the affected side. Few previous studies calculated orbital volume and orbital volume ratios (ipsi- to contralateral side) in patients with UCS. Beckett et al. [3] found a mean ratio of 93.8 (*N* = 21; unclear pre- or postoperative; mean age 5.5 months), Bentley et al. [5] reported a mean ratio of 92.0 (*N* = 12; preoperative; age 1 to 29 months (82% within the 1st year of life)). Only one study focused on linking orbital volumes to severity of UCS. Calandrelli et al. [7] categorised the patients with UCS (*N* = 24; unclear pre- or postoperative; mean age 162 days (90–256 days)) according to the skull base classification method by Di Rocco et al. [9], resulting in ratios of 92.0 (groups IIA and IIB; moderate) and 91.0 (group III; severe). They found a trend in progressively reducing volumes on the affected side according to the severity of the group, but no statistical significant correlation.

The orbital volume ratios in patients with UCS found in our study are comparable to those found in other studies. We did not find an association between severity of UCS, according to UCSQ, and orbital volume ratio. Mean orbital volume ratio in both the mild and moderate group was 0.92, and mean orbital volume ratio in the severe group was 0.93. One could expect a negative correlation between severity of UCS and orbital volume ratio, since a more severe form of UCS leads to a visually more asymmetric skull shape. However, in the present study, a more severe UCS did not correlate with a smaller orbital volume ratio. We did not compare and correlate absolute values of orbital volume on the affected side to severity of UCS. We believe orbital volume ratio, and therefore the ratio between affected and unaffected orbit is more indicative for severity of consequences of UCS than an absolute value. Additionally, by calculating ratios, we are able to compare children of different ages (months) included in this study.

Our subsequent aim was to link orbital volume and severity of UCS to ophthalmic sequelae. UCS has effects on ocular motility through the changes in shape and axis of the orbit on the synostotic side. The bony deformation in the frontozygomatic region can result in traction on the ocular globe [8]. This direct traction of this region on the lateral check ligament of the lateral rectus muscle causes stretching of the lateral rectus unilaterally in UCS [26]. The stretching results in an increased passive tone of the ocular muscles and an increased extraocular muscle tone from less efficient orbital movements, possibly resulting in strabismus and abnormal extraocular motility [30]. Also, the orbital deformity (Harlequin orbit), results in an abnormal pulley location of the superior oblique, mimicking a weakness of the superior oblique and leading unopposed action of the inferior oblique muscles, resulting in abnormal extraocular motility and strabismus [1, 8, 23, 27, 32].

We reported impairment of eye movement in 10.5% of patients (2/19); one abduction impairment (on both the ipsi- and contralateral side) and one adduction impairment (ipsilateral side). Our found prevalence is lower than found in literature: limitation of eye movement was reported preoperatively in 54% (32/59; 82% age < 2 years) [23].

The prevalence of strabismus (misalignment of the eyes) is reported by Friedman et al.: 1% following ophthalmic screening of 38.000 healthy infants (age 1 to 2.5 years) [11]. Our study showed strabismus in 10.5% of patients (2/19; 1 vertical and 1 not mentioned). Several other studies collected strabismus preoperatively in UCS patients; strabismus is noted in 64% (9/14; median age at surgery 9.5 months), 55% (6/11; mean age 7 months) and 58% (34/59; 82% age < 2 years) [12, 23, 31]. However, it is notable that in the latter study, 46% (19/34) of strabismus occurs on the contralateral eye, 27% (9/34) on the ipsilateral eye and 18% (6/34) on alternating eyes [23].

We did not find amblyopia in the included patients. A prevalence of amblyopia of 1% following ophthalmic screening of 38.000 healthy infants (age 1 to 2.5 years) was reported [11]. In pre- and postoperative UCS patients, amblyopia is found in 38% (15/39; in 12/15 (80%) on contralateral eye; median age 1.5 years (3 months to 28 years)) [22].

A percentage of 25% (126/514 healthy children; age 1 to 48 months) of healthy children with astigmatism was reported [25]. We found astigmatism in 5% of patients (2/37). A study with both pre- and postoperative UCS patients reported astigmatism noted in 54% (21/39; median age 1.5 years (3 months to 28 years)) [22]. Additionally, astigmatism was found in 29% (2/7; mean age 13 months (SD 22 months); unclear whether pre- or postoperative patients) in another study [14].

We did not report preoperative visual field asymmetry, preoperative visual field asymmetry was recorded in 45% (5/11; mean age 7.5 months) of the UCS patients [31].

In general, caution should be taken when comparing our ophthalmic results with the existing literature, as the ages of included patients vary widely in literature, and pre- and postoperative patients are mixed into one patient group. The present study only includes preoperative UCS patients in order to analyse ophthalmic sequelae and evaluate the influence of orbital volume asymmetry. Hereby, only the effect of the fused suture on ophthalmic complications is analysed and not the effect of surgery. In most of the UCS surgeries, the orbit is part of the surgical field and thereby ophthalmic results will be influenced (to some extent) by surgery. By using this clean group of patients, the patients are inevitable young and therefore it is difficult to diagnose and objectify ophthalmic sequelae, possibly leading to an underestimation of the prevalence of the described complications preoperatively. Therefore, it remains important to keep the young patients with UCS under precise orthoptic and ophthalmologic examinations, independent of the severity of UCS. Small manifest squints have the same inherent effect of visual loss through amblyopia as larger squints, and clinicians need to be aware that this can occur on the nonsynostotic side as well as the synostotic side, and the patient should be kept under close regular monitoring both pre- and postoperatively [23]. Additionally, it should be noted that our sample size is relatively small and ophthalmic sequealae only occurred in three of the 19 patients.

We found side-to-side asymmetry between the orbits on the affected and unaffected side, with a reduced orbital volume on the affected side. No association between severity of UCS according to UCSQ and orbital volume ratio was found. No association between either preoperative orbital volume ratio or severity of UCS and the presence of preoperative ophthalmic sequelae was found. Additionally, no association between orbital index, proptosis, presence of deviation of nasal root and angulation of the sphenoid ridge and ophthalmic sequelae was found.

## Data Availability

Not applicable

## References

[1] Bagolini B, Campos EC, Chiesi C (1982). Plagiocephaly causing superior oblique deficiency and ocular torticollis. A new clinical entity. Arch Ophthalmol.

[2] Baranello G, Vasco G, Ricci D, Mercuri E (2007). Visual function in nonsyndromic craniosynostosis: past, present, and future. Childs Nerv Syst.

[3] Beckett JS, Persing JA, Steinbacher DM (2013). Bilateral orbital dysmorphology in unicoronal synostosis. Plast Reconstr Surg.

[4] Benson ML, Oliverio PJ, Yue NC, Zinreich SJ (1996). Primary craniosynostosis: imaging features. AJR Am J Roentgenol.

[5] Bentley RP, Sgouros S, Natarajan K, Dover MS, Hockley AD (2002). Changes in orbital volume during childhood in cases of craniosynostosis. J Neurosurg.

[6] Bruneteau RJ, Mulliken JB (1992). Frontal plagiocephaly: synostotic, compensational, or deformational. Plast Reconstr Surg.

[7] Calandrelli R, Pilato F, Massimi L, Panfili M, Di Rocco C, Colosimo C (2018). Quantitative analysis of cranial-orbital changes in infants with anterior synostotic plagiocephaly. Childs Nerv Syst.

[8] Denis D, Genitori L, Conrath J, Lena G, Choux M (1996). Ocular findings in children operated on for plagiocephaly and trigonocephaly. Childs Nerv Syst.

[9] Di Rocco C, Paternoster G, Caldarelli M, Massimi L, Tamburrini G (2012). Anterior plagiocephaly: epidemiology, clinical findings,diagnosis, and classification. A review. Childs Nerv Syst.

[10] Domeshek LF, Woo A, Skolnick GB, Naidoo S, Segar D, Smyth M, Proctor M, Patel KB (2019). Postoperative changes in orbital dysmorphology in patients with unicoronal synostosis. J Craniofac Surg.

[11] Friedman Z, Neumann E, Hyams SW, Peleg B (1980). Ophthalmic screening of 38,000 children, age 1 to 2 1/2 years, in child welfare clinics. J Pediatr Ophthalmol Strabismus.

[12] Gosain AK, Steele MA, McCarthy JG, Thorne CH (1996). A prospective study of the relationship between strabismus and head posture in patients with frontal plagiocephaly. Plast Reconstr Surg.

[13] Gribova MN, Pluijmers BI, Resnick CM, Caron CJJM, Borghi A, Koudstaal MJ, Padwa BL (2018). Is there a difference in orbital volume between affected and unaffected sides in patients with unilateral craniofacial microsomia. J Oral Maxillofac Surg.

[14] Gupta PC, Foster J, Crowe S, Papay FA, Luciano M, Traboulsi EI (2003). Ophthalmologic findings in patients with nonsyndromic plagiocephaly. J Craniofac Surg.

[15] Haaga JR, Boll D (2009). CT and MRI of the whole body.

[16] Kahilogullari G, Uz A, Eroglu U, Apaydin N, Yesilirmak Z, Baskaya MK, Egemen N (2012). Does the sphenoid angle effect the operation strategy? Anatomical and radiological investigation. Turk Neurosurg.

[17] Kotrikova B, Krempien R, Freier K, Mühling J (2007). Diagnostic imaging in the management of craniosynostoses. Eur Radiol.

[18] Kronig SAJ, Kronig ODM, Vrooman HA, Veenland JF, Van Adrichem LNA (2020) Quantification of severity of unilateral coronal synostosis. Cleft Palate Craniofac J:105566562096509. 10.1177/1055665620965099 10.1177/1055665620965099 PMC8209757 33078622

[19] Kronig ODM, Kronig SAJ, Vrooman HA, Veenland JF, Jippes M, Boen T, Van Adrichem LNA (2020). Introducing a new method for classifying skull shape abnormalities related to craniosynostosis. Eur J Pediatr.

[20] Kronig SAJ, Kronig ODM, Vrooman HA, Veenland JF, Van Adrichem LNA (2020) New diagnostic approach of the different types of isolated craniosynostosis. Eur J Pediatr. 10.1007/s00431-020-03860-9 10.1007/s00431-020-03860-9 PMC7940292 33151409

[21] Lee SJ, Dondey J, Greensmith A, Holmes AD, Meara JG (2008). The effect of fronto-orbital advancement on strabismus in children with unicoronal synostosis. Ann Plast Surg.

[22] Levy RL, Rogers GF, Mulliken JB, Proctor MR, Dagi LR (2007). Astigmatism in unilateral coronal synostosis: incidence and laterality. J AAPOS.

[23] Macintosh C, Wall S, Leach C (2007). Strabismus in unicoronal synostosis: ipsilateral or contralateral. J Craniofac Surg.

[24] Marsh JL, Gado MH, Vannier MW, Stevens WG (1986). Osseous anatomy of unilateral coronal synostosis. Cleft Palate J.

[25] Mayer DL, Hansen RM, Moore BD, Kim S, Fulton AB (2001). Cycloplegic refractions in healthy children aged 1 through 48 months. Arch Ophthalmol.

[26] McCarthy JG, Coccaro PJ, Eptstein F, Converse JM (1978). Early skeletal release in the infant with craniofacial dysostosis: the role of the sphenozygomatic suture. Plast Reconstr Surg.

[27] Morax S (1984). Oculo-motor disorders in craniofacial malformations. J Maxillofac Surg.

[28] Nout E, van Bezooijen JS, Koudstaal MJ, Veenland JF, Hop WC, Wolvius EB, van der Wal KG (2012). Orbital change following Le Fort III advancement in syndromic craniosynostosis: quantitative evaluation of orbital volume, infra-orbital rim and globe position. J Craniomaxillofac Surg.

[29] Portney LG, Watkins MP (2000). Foundations of clinical research: applications to practice.

[30] Read SA, Collins MJ, Carney LG (2007). A review of astigmatism and its possible genesis. Clin Exp Optom.

[31] Ricci D, Vasco G, Baranello G, Salerni A, Amante R, Tamburrini G, Dickmann A, Di Rocco C, Velardi F, Mercuri E (2007). Visual function in infants with non-syndromic craniosynostosis. Dev Med Child Neurol.

[32] Robb RM, Boger WP (1983). Vertical strabismus associated with plagiocephaly. J Pediatr Ophthalmol Strabismus.

[33] Watts GD, Antonarakis GS, Blaser SI, Phillips JH, Forrest CR (2019). Cranioorbital Morphology Caused by Coronal Ring Suture Synostosis. Plast Reconstr Surg.

